# Corneal ectasia following photorefractive keratectomy: a confocal microscopic case report and literature review

**DOI:** 10.5935/0004-2749.2021-0296

**Published:** 2023-03-20

**Authors:** Azam Alvani, Hassan Hashemi, Mohammad Pakravan, Mohammad Reza Aghamirsalim

**Affiliations:** 1 Noor Ophthalmology Research Center, Noor Eye Hospital, Tehran, Iran; 2 Noor Research Center for Ophthalmic Epidemiology Noor Eye Hospital, Tehran, Iran; 3 Ophthalmic Epidemiology Research Center, Shahid Beheshti University of Medical Sciences, Tehran, Iran; 4 Translational Ophthalmology Research Center, Tehran University of Medical Sciences, Tehran, Iran

**Keywords:** Cornea/pathology, Dilatation, pathologic, Keratoconus, Photorefractive keratectomy, Keratomileusis, laser in situ, Microscopy, confocal, Humans, Case reports, Córnea/patologia, Dilatação patológica, Ceratoconus, Ceratectomia fotorrefrativa, Ceratomileuse assistida por excimer laser in situ, Microscopia confocal, Humanos, Relato de casos

## Abstract

The occurrence of corneal ectasia after photorefractive keratectomy is a rare but serious complication of refractive surgery. Possible risk factors are not well assessed, but a probable reason is the failure to detect keratoconus preoperatively. In this report, we describe a case of corneal ectasia after photorefractive keratectomy in a patient who presented a suspicious tomography pattern preoperatively but had no degenerative alterations associated with pathologic keratoconus, as revealed by in vivo corneal confocal microscopy. We also review eligible case reports of post-photorefractive keratectomy ectasia to find similar characteristics.

## Abstract (pt)

A ocorrência de ectasia corneana após ceratectomia fotorrefrativa é uma complicação rara, porém grave, em cirurgia refrativa. Os possíveis fatores de risco não são bem avaliados, mas a opinião atual é que a falha na detecção de ceratocone pré-operatório possa ser o principal motivo. Neste relato, descrevemos um caso de ectasia corneana após ceratectomia fotorrefrativa em paciente apresentando padrão tomográfico suspeito no pré-operatório, mas sem alterações degenerativas associadas a ceratocone patológico, conforme revelado por microscopia confocal in vivo da córnea. Além disso, revisamos, na literatura, relatos de casos elegíveis de ectasia pós-ceratectomia fotorrefrativa para encontrar características semelhantes.

## INTRODUCTION

Corneal ectasia is a rare but serious complication of refractive surgery. It occurs more frequently following laser-assisted in situ keratomileusis (LASIK)^(1)^, and some studies have suggested performing photorefractive keratectomy (PRK) instead of LASIK to prevent ectasia in predisposed corneas. However, some cases of post-PRK ectasia were reported^(2,3,4,5,6,7,8)^. Although risk factors are unknown, it is widely thought that keratoconus undiagnosed preoperatively is the primary cause.

Herein, we describe the clinical and microstructural features of a patient with a borderline suspicious tomography pattern and normal diagnostic indices who underwent bilateral PRK and developed ectasia in both corneas 7 years later. Additionally, all case reports of post-PRK ectasia with documented preoperative topography were evaluated for similar characteristics.

## CASE REPORT

**Figure 1:**
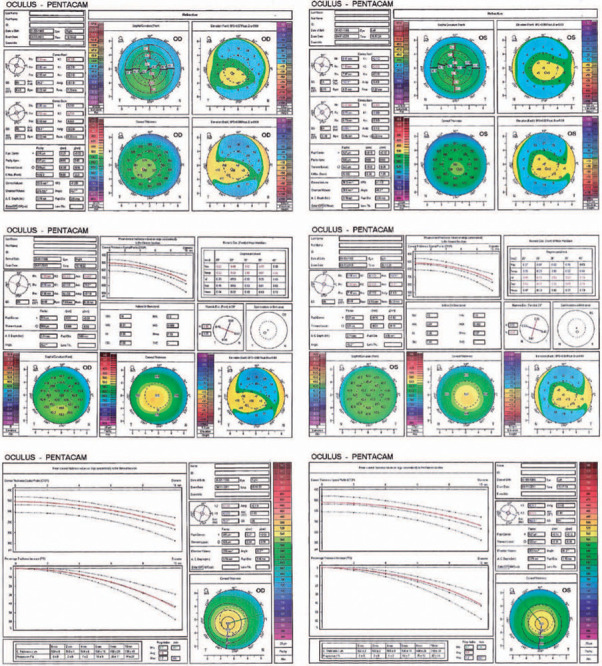
Preoperative corneal tomography images in both eyes.

A 25-year-old man who had no keratoconus signs on slit-lamp biomicroscopy and retinoscopy and had a negative familial history of keratoconus underwent uneventful PRK with Concerto 500Hz Excimer Laser (Wavelight AG, Erlangen, Germany) in both eyes in June 2011. The preoperative cycloplegic refractions were -4.5 -0.75 × 85 and -6.75 -0.5 × 120 in the right and left eyes, respectively, and the corrected distance visual acuity (CDVA) was 20/20 in both eyes. In the measurement with Pentacam HR (OCULUS, Inc., Wetzlar, Germany), the elevation numbers of the anterior surface of both eyes and posterior surface of the left eye were within normal range, while in the posterior surface of the right eye, one point had +21 µm elevation. The sagittal map of the anterior corneal surface showed a slight inferior steepening in the right eye and slight with-the-rule astigmatism in the left eye. The simulated keratometry values of the front surface of the cornea were 42.2/42.3 and 42.1/42.6 diopter in the right and left eyes, respectively. In the right eye, the central corneal thickness (CCT) was 517 µm (thinnest point [TP] = 509 µm), and the TP displacement relative to the center was -0.56 mm. In the left eye, the CCT was 526 µm (TP=522 µm), and TP displacement relative to the center was -0.7 mm. Keratoconus and corneal thickness profile indices were all normal in both eyes (Figure 1).

**Figure 2:**
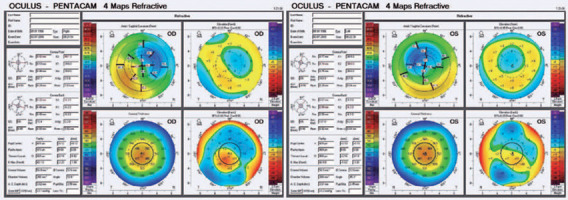
Postoperative corneal tomography images 7 years later, indicating corneal ectasia in both eyes.

The patient underwent surgery with an optical zone of 6 mm and maximum stromal ablation depths of 68.86 and 86.37 µm in the right and left eyes, respectively. Six months after the operation, the manifest refraction was Plano -0.5 × 75 and -0.25 diopter sphere in the right and left eyes, respectively, which remained stable for 5 years. In May 2017, the patient presented with blurry vision and manifest refraction of -1.00 -0.75 × 95 and -0.5 -0.25 × 65 in the right and left eyes, respectively. The patient was diagnosed with myopia regression and provided the required glasses to correct vision. The patient returned for refractive surgery 14 months later. The result of cycloplegic refraction at this time was -1.00 -1.00 × 90 in the right eye and -1.00 -0.25 × 120 in the left eye, and CDVA was 20/20 in both eyes. Pentacam sagittal map showed an inferior temporal steepening indicating corneal ectasia in both eyes, which was more severe in the right eye (Figure 2).

**Figure 3:**
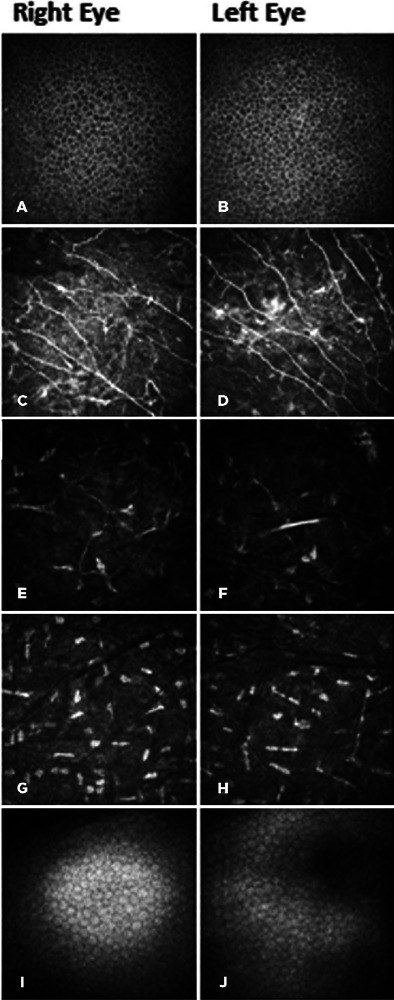
400 × 400 µm in vivo confocal microscopy images, illustrating the basal epithelium (A, B), hyperreflective sub- basal nerve fibers (C, D), anterior stromal keratocytes (E, F), posterior stromal keratocytes (G, H), and endothelium (J, K) in right and left eyes 7 years after photorefractive keratectomy.

The patient was sent to our service based on the diagnosis for an in vivo confocal microscopy (IVCM) (HRT III-RCM, Heidelberg Engineering GmbH, Dossenheim, Germany) assessment of corneal microstructures. The test was performed in the sequence mode on the central cornea. After selecting two images with the highest quality for each layer of the basal epithelium, anterior keratocyte, posterior keratocyte, and endothelium, the cell density was calculated using the software embedded in HRT III-RCM. A standard central counting frame size of 200 × 200 µm^2^ was considered for basal epithelium and endothelium images and 300 × 300 µm^2^ for anterior and posterior keratocyte images. The number of cells per mm^2^ was measured in each image, and the average of two measurements was calculated.^(9,10)^ Sub-basal nerve (SBN) fiber density was measured using automated CCMetrics (ACCMetrics) software version 1.0 (University of Manchester, UK).^(11)^ The results showed a lack of Bowman’s layer, hyperreflectivity of SBN fibers, and reduction of the most anterior keratocytes in both eyes (123 and 174 cells/mm^2^ in right and left eyes, respectively). The density of the basal epithelial cells (BECs) (6055 and 7004 cells/mm^2^ in the right and left eyes, respectively), endothelial cells (3247 and 2951 cells/mm^2^ in the right and left eyes, respectively), and SBN fibers (25 and 37.5 nerves/mm^2^ in the right and left eyes, respectively) were normal. The reduction of the density of the most posterior keratocytes was not noticeable (301 and 228 cells/mm^2^ in right and left eyes, respectively) (Figure 3).

## DISCUSSION

A review of the literature identified seven case reports^(2,3,4,5,6,7,8)^ with documented preoperative topography results, reporting a total of 11 post-PRK ectasia cases (Table 1). In all reported cases^(2,4,5,6,7,8)^, except for one^(3)^, the same eye or fellow eye was diagnosed with clinical keratoconus, forme fruste keratoconus, or keratoconus suspect preoperatively, or there was a positive familial history of keratoconus, or the corneal thickness was ≤520 µm. In the reports by Malecaze et al.^(2)^ and Navas et al,^(6)^ forme fruste keratoconus was considered equivalent to keratoconus suspect, and in reports by Leccisotti^(5)^ and Bardocci et al.^(8)^, the diagnosis of keratoconus suspect was made regardless of the findings of slit-lamp biomicroscopy and retinoscopy, which may be due to the unavailability of complete preoperative data. In all previous reports with the diagnosis of forme fruste/ suspected keratoconus, significant topographic abnormalities, sometimes accompanied by a positive familial history of keratoconus, may suggest early keratoconus. However, these cases are now easily detectable, and surgeons are usually cautious enough in dealing with such patients. The main challenge is in patients who are candidates for refractive surgery without any clinical signs or familial history of keratoconus, but with a borderline suspicious topography/tomography pattern, as in the presented case.

**Table 1:** Preoperative data of previously reported post-photorefractive keratectomy ectasia cases

Authors		Patient’s sex, age, affected eye, CDVA, CCT, and ectasia detection time	Refraction (Diopter)	Keratometry (Diopter)	Topography	Other findings
**Malecaze et al**^(2)^ **(2005)**		Male, 22 y, OU, OU: 20/20, OU: 495 µm, 4 y later	OD: -1.50 -1.00 × 105 OS: -1.50 -1.00 × 65	OD: 43.75/43.32 OS: 43.88/43.26	OD: moderate temporal steepening, SRAX = 37, KISA% = 12.3 OS: inferior steepening, J shape, SRAX = 60, I-S value = 0.9, KISA% = 20 **Final diagnosis: FFKC (OS)**	Normal slit-lamp biomicroscopy findings, negative familial history
**Chiou et al**^(3)^ **(2006)**		Male, 40 y, OU, OU: 20/25, OD: 477 µm / OS: 481 µm, 18 months later	OD: -9.25 -1.25 × 65 OS: -8.25 -2.00 × 160	-	Normal	Post-PRK severe corneal haze and long-term steroid use, severe corneal tissue ablation
**Randleman et al**^(4)^ **(2006)**	**Case 1**	Male, 37 y, OU, OD: 20/20/ OS: 20/30, OD: 472 µm / OS: 441 µm, 2 weeks later	OD: -1.50 -2.50 × 70 OS: -4.00 -3.00 × 90	OD: 45.50/46.50 OS: 48.50/50.20	KC in both eyes	-
	**Case 2**	Male, 40 y, OU, OD: 20/20^-2^/ OS: 20/20^-1^, OD: 509 µm / OS: 508 µm, 10 months later	OD: -5.75 -3.75 × 33 OS: -5.25 -4.00 × 167	OD: 46.68/43.21 OS: 46.80/43.38	Symmetric bowtie, high oblique astigmatism	Normal slit-lamp biomicroscopy findings, positive familial history, postoperative IOP rising, and eye rubbing
**Leccisotti**^(5)^ **(2007)**	**Case 1**	Female, 38 y, OU, -, OD: 520 µm / OS: 510 µm, 3 y later	OD: -7.00 -3.00 × 180 OS: -6.00 -4.00 × 180	OD: 44.30 OS: 45.50	High astigmatism and inferior steepening (1.3 and 1.6 D) in both eyes **Final diagnosis: KC in both eyes**	-
	**Case 2**	Male, 31 y, OD, 20/20, 487 µm, 1 y later	OD: -4.50 -1.75 × 175	OD: 45.75	KC in the fellow eye (OS)	-
	**Case 3**	Male, 31 y, OS, 20/20, OD: 506 µm / OS: 492 µm, 5 months later	OS: -3.75 -0.50 × 150	OS: 45.70	Evident inferior steepening, asymmetric bowtie with SRAX in both eyes, abnormal ABR index in OS. Videokeratography ruled out KC in both eyes. **Final diagnosis: KC suspect (OS)**	No information about familial history of KC, slit-lamp biomicroscopy findings, or retinoscopy
	**Case 4**	Female, 38 y, OS, 20/20, 509 µm, 5 months later	OS: -1.50 -1.25 × 130	OS: 48.30	Evident inferior steepening especially in OS, abnormal vertical asymmetry index, and Kmax = 48.30 in OS. Videokeratography ruled out KC in OD and defined possible in OS. **Final diagnosis: KC suspect (OS)**	No information about familial history of KC, slit-lamp biomicroscopy findings, or retinoscopy
**Navas et al**^(6)^ **(2007)**		Male, 35 y, OU, OU: 20/20, OD: 497 µm / OS: 511 µm, 2 weeks later	OD: -3.00 -1.50 × 20 OS: -3.00 -2.00 × 160	OD: 43.25/45.00 OS: 43.25/45.50	Asymmetric astigmatism and FFKC in both eyes which was more pronounced in OD	Normal slit-lamp biomicroscopy findings, positive familial history, eye rubbing
**Reznik et al**^(7)^ **(2008)**		Male, 25 y, OD, OU: 20/25, OD: 500 µm / OS: 460 µm, 5 y later	OD: -5.75 -1.75 × 95 OS: -7.50 -1.25 × 80	OD: 42.50/42.25 OS: 43.25/44.00	KC (OD)	-
**Bardocci et al**^(8)^ **(2012)**		Female, 31 y, OU, -, OD: 512 µm / OS: 520 µm, 6 y later	OD: -8.00 -2.00 × 30 OS: -4.50 -1.50 × 150	-	AB/SRAX in OD, D pattern in OS **Final diagnosis: KC suspect (OU)**	No information about familial history of KC, personal history of allergy or eye rubbing, slit-lamp biomicroscopy findings, or retinoscopy

ABR= aberration coefficient; CCT= central corneal thickness; CDVA= corrected distance visual acuity; FFKC= forme fruste keratoconus; I-S value= inferior/superior value; KC= keratoconus; KISA%= keratoconus percentage index; Kmax= maximum keratometry; OD= oculus dexter (right eye); OS= oculus sinister (left eye); OU= oculus uterque (both eyes); PRK= photorefractive keratectomy; SRAX= skewed radial axis; Y= years.

Alterations in corneal microstructures, including decreased BECs, anterior and posterior stromal keratocytes, and SBN fiber density, have been reported in patients with keratoconus^(9,10)^. Considering the results of Bitirgen et al.^(9)^ in normal virgin corneas, BECs, endothelial cells, and SBN fiber density in the present case appeared to be normal. The reduction of posterior keratocytes was not remarkable. However, a considerable reduction in the density of keratocytes in the most anterior stromal layer was found. These results corroborate previously published findings^(12,13,14)^ on normals patient post-PRK. Long-term studies conducted 5, 10, and 20 years after PRK have shown no difference in the density of BEC, SBNs, posterior keratocytes, and the density and morphology of endothelial cells between normal patients post-PRK and controls^(12,14)^. However, the anterior keratocyte density was 47% lower than the preoperative levels at 5 years follow-up^(13)^. By contrast, the IVCM results in our case are distinct from those associated with pathological keratoconus, which are related to quantitative and qualitative changes in BECs and SBN fibers^(9,10)^. In our previous report on patients with keratoconus and post-LASIK ectasia, contrary to the differences of the keratoconus group with their normal virgin controls, the post-LASIK ectasia group had no difference with their normal post-LASIK controls in terms of BECs, anterior and posterior stromal keratocytes, endothelium, and SBN fiber density^(10)^. However, compared with normal virgin controls, both post-LASIK controls and post-LASIK ectasia groups showed a similar reduction in the levels of the anterior and posterior keratocyte density^(10)^. In the mentioned study^(10)^, based on the findings, we concluded that, contrary to the degenerative nature of keratoconus, post-LASIK ectasia can have a mechanical nature. However, LASIK differs from PRK in terms of flap creation, depth of stromal ablation, and severity of damage to corneal innervation. This may explain why the current case had a lesser drop in posterior keratocyte density than the post-LASIK ectasia group in our previous report.

To the best of our knowledge, this is the first IVCM report of a post-PRK ectasia, which showed that post-PRK ectasia could also develop in corneas with a borderline topographic pattern without keratoconus-related cellular changes. Although some studies have shown that PRK in patients with bilateral asymmetric topography or even in eyes with suspected keratoconus may be performed^(15,16)^, some mechanical stimuli can still initiate the ectatic process in these corneas^(17)^. The delayed corneal protrusion in our case may be explained by the effect of normal intraocular pressure (IOP) on a biomechanically weakened cornea^(18)^. Based on a recently introduced theory, ectasia is the thinning and protrusion of a localized biomechanically weak corneal area under IOP load over time, which gradually develops to surrounding areas^(18)^. An association between keratoconus and conditions such as floppy eyelid, obstructive sleep apnea, and ocular allergy has been reported in some studies^(19,20,21)^ but our patient did not have any of these conditions. However, eye rubbing and sleeping with eyes squeezed against the pillow should be recognized as two probable causes of ectasia over time.^(17,20,21,22,23)^

Based on the results of the present case and literature review, an abnormal preoperative topography is an important risk factor for the development of corneal ectasia following PRK. Refractive surgeons should avoid refractive surgery, including surface ablation procedures such as PRK in such cases, particularly if there is any possibility of a mechanical stimulation causing additional corneal biomechanical weakening. In addition, we recommend considering preoperative corneal confocal microscopy in borderline cases indicated for PRK when it is available to rule out any abnormalities in corneal cells or SBN fibers related to keratoconus and establish a comparison if these eyes develop ectasia later. This may extend our knowledge of corneal ectasia formation following PRK.
